# Chemopreventive Activity of Vitamin E in Breast Cancer: A Focus on γ- and δ-Tocopherol

**DOI:** 10.3390/nu3110962

**Published:** 2011-11-14

**Authors:** Amanda K. Smolarek, Nanjoo Suh

**Affiliations:** 1 Department of Chemical Biology, Ernest Mario School of Pharmacy, 164 Frelinghuysen Road, Rutgers, The State University of New Jersey, Piscataway, NJ 08854, USA; Email: smoaman@eden.rutgers.edu; 2 Joint Graduate Program in Toxicology, Rutgers, The State University of New Jersey, Piscataway, NJ 08854, USA; 3 The Cancer Institute of New Jersey, New Brunswick, NJ 08901, USA

**Keywords:** vitamin E, tocopherols, breast cancer, estrogen receptor (ER), peroxisome proliferator activated receptor γ (PPARγ), nuclear factor (erythroid-derived 2)-like 2 (Nrf2), anti-inflammatory, cell proliferation, apoptosis, case-control studies

## Abstract

Vitamin E consists of eight different variants: α-, β-, γ-, and δ-tocopherols (saturated phytyl tail) and α-, β-, γ-, and δ-tocotrienols (unsaturated phytyl tail). Cancer prevention studies with vitamin E have primarily utilized the variant α-tocopherol. To no avail, a majority of these studies focused on variant α-tocopherol with inconsistent results. However, γ-tocopherol, and more recently δ-tocopherol, have shown greater ability to reduce inflammation, cell proliferation, and tumor burden. Recent results have shown that γ-enriched mixed tocopherols inhibit the development of mammary hyperplasia and tumorigenesis in animal models. In this review, we discuss the possible differences between the variant forms, molecular targets, and cancer-preventive effects of tocopherols. We recommend that a γ-enriched mixture, γ- and δ-tocopherol, but not α-tocopherol, are promising agents for breast cancer prevention and warrant further investigation.

## 1. Tocopherols

**Figure 1:**
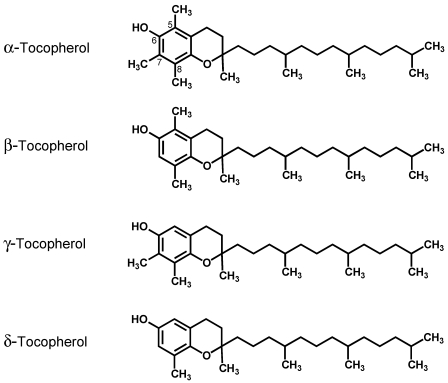
Chemical structures of α-, β-, γ-, and δ-tocopherols.

Due to its antioxidant properties, dietary intake of vitamin E, a fat-soluble vitamin, has been suggested to reduce cancer risk [1]. Vitamin E consists of eight different forms which include four tocopherols (with a saturated phytyl tail) and four tocotrienols (with an unsaturated isoprenoid side chain), designated as α, β, γ, and δ variants (Figure 1) [2]. 

α-Tocopherol is known as the “classic” vitamin E, because of its superior activity over the other tocopherols in the classic fertility restoration assay [3]. α-Tocopherol is most commonly found in wheat germ, almond, and sunflower oil [4]. However, γ-tocopherol is more prominent than α-tocopherol in the American diet and is found in vegetable oils such as soybean, corn, and cottonseed [5]. δ-Tocopherol is primarily found in soybean and castor oils, and to a lesser extent, in wheat germ oil [6]. A tocopherol mixture containing 58% γ-tocopherol, 24% δ-tocopherol, 13% α-tocopherol, and 0.5% β-tocopherol (γ-TmT) can be easily available as a by-product of refining vegetable oil [7,8]. Tocotrienols are consumed more readily in East-South Asian diets, and found primarily in palm and annatto oils [9,10]. Since tocopherols are the main components of vitamin E in the American diet, this review will focus on tocopherols. The first non antioxidant function of vitamin E determined that α-tocopherol inhibited the activity of smooth muscle proliferation and protein kinase C [11,12]. Since then, three proteins have been identified to specifically bind to tocopherols: α-tocopherol transfer protein (α-TTP), tocopherol-associated protein (TAP), and tocopherol-binding protein (TBP). α-TTP is a 30-35 kDa protein and found in the liver [13] which preferentially transfers α-tocopherol from the liver to the blood [14]. The relative affinities of α-TTP for the variants of vitamin E as determined *in vitro* were 100% for α-tocopherol, 38% for β-tocopherol, 9% for γ-tocopherol, 2% for δ-tocopherol, and 12% for α-tocotrienol [15]. Thus, the major tocopherol found in human blood and tissues is α-tocopherol [3]. Similar to α-TTP, TAP is also a cystolic lipid-binding and transfer protein. TAP is a 46-kDa protein and has the highest levels in the liver > prostate > whole brain > spinal cord > kidney > mammary gland > stomach [16]. TBP was initially found in rat liver and heart is an approximately 15 kDa cystolic protein [17] and later in human placenta [18]. TBP is involved in intracellular transport and metabolism for α-tocopherol [19]. 

In the liver, vitamin E is metabolized to chromanol metabolites via the hepatic protein, cytochrome P450 4F2. CYP4F2 catalyzes the initial step in the vitamin E-ω-hydroxylase pathway followed by β-oxidation, which removes 2 carbons from the side chain in each cycle ending in the short chain metabolite, carboxyethyl hydroxychromans (CEHC) [14,20]. Since α-tocopherol is preferentially transferred to the blood by α-TTP, γ-tocopherol and δ-tocopherol are more readily metabolized in the liver [14]. 

Interestingly, higher concentrations of α-tocopherol may decrease the level of γ-tocopherol in the serum [21,22]. This may be unfavorable since γ-tocopherol has demonstrated significantly greater anti-inflammatory and anti-tumor activity than α-tocopherol in several different animal models of colon, breast, and prostate cancer [22,23,24,25,26,27]. More specifically, γ-tocopherol is more effective in inhibiting the activity of cyclooxygenase-2 (COX-2) [23,28] and trapping reactive nitrogen species than α-tocopherol [23,28,29,30,31,32]. 

The stability of tocopherol and nitrogen species derivative depends on the structure of the chromanol ring [32]. The tocopherols with a free 5 position on the chromanol ring (γ- and δ-tocopherol) are expected to react with nitrogen species forming C-nitroso derivatives at this position [32]. Both α-tocopherol and γ-tocopherol react with nitrogen dioxide NO_2_; α-tocopherol forms an intermediate tocopheroxide analogue while γ-tocopherol may form nitric oxide (NO) or a stable nitro derivative (5-nitro-γT) [32]. α-Tocopherol is trimethylated, and consequently, the nitrosating agent only has the possibility to add to the *para*-position on the chromanol ring of α-tocopherol, forming a highly unstable compound and may form toxic *N*-nitroso-derivatives from amines [32]. In addition, α-tocopherol may react with nitrous acid to yield α-tocopherol quinone and nitrogen monoxide gas [33]. This may lead to highly instable derivatives which may act as nitrosation catalysts for secondary amines. The high hydrogen donation ability by α-tocopherol may cause undesirable side effects, such as pro-oxidant and toxic nitro derivatives [34]. 

Tocopherols are recognized for their inhibition of lipid oxidation [35]. The antioxidant properties are mostly due to the phenolic hydrogens in the chromanol ring that are donated to lipid free radicals [36]. α-Tocopherol is trimethylated at the 5-, 7-, and 8-positions on the chromanol ring, γ-tocopherol is dimethylated at 7- and 8-positions, and δ-tocopherol is monomethylated at the 8-postion on the chromanol ring. The structural difference in the chromanol ring may be responsible for the difference in activity of each individual tocopherol form. The *ortho*-positions (positions 5 and 7) for the methyl groups on the chromanol ring enhance the antioxidant properties of tocopherols and increases the solubility in lipid substrates [33]. Thus, α-tocopherol with two *ortho*-methyl groups is expected to be a more potent hydrogen donor than either γ-tocopherol (one *ortho* methyl group) and δ-tocopherol (zero *ortho* methyl group) [33]. Although α-tocopherol may be a better antioxidant, α-tocopherol consequently has a greater capacity than γ-tocopherol and δ-tocopherol to act as a prooxidant when present in high concentrations in vegetable oils, and with transition metal ions, lipid peroxides, and other oxidizing agents [33,34].

α-Tocopherol has been the most widely studied form of vitamin E for the prevention and treatment of cancer [37,38,39,40]. Although the biological effects of α-tocopherol have been investigated over many decades, our current understanding of its role in inhibiting breast carcinogenesis remains incomplete [41]. The structural difference of the individual tocopherols plays a role in the variance of antioxidant properties, lipophilicity, and the ability to trap reactive nitrogen species (RNS). Both γ- and δ-tocopherol, but not α-tocopherol, show promise as chemopreventive agents in animal models [7,42,43]. In addition, γ-TmT is a mixture of tocopherols enriched with γ-tocopherol and is readily available and inexpensive, while individual variants remain expensive to purify. As a result, γ-TmT may be more practical rather than individual tocopherols for the prevention of breast cancer. 

## 2. Subtypes of Breast Cancer

Breast cancer is one of the most common malignancies affecting women and is the second leading cause of cancer death in women [44]. The etiology and pathogenesis of breast cancer remains poorly understood. Breast cancer is a heterogeneous disease that can be classified into subtypes based on immunohistochemical markers. The subtypes are: estrogen receptor (ER) positive luminal A, ER positive luminal B, human epidermal growth factor receptor-2 positive (HER2 positive), and basal-like [45]. 

### 2.1. Estrogen Receptor (ER) Positive

Estrogen receptor positive tumors are classified as a luminal subtype of breast cancer and are reported in 60-70% of cases [45]. Luminal tumors activate ER-responsive genes, other genes that encode characteristic proteins of luminal epithelial cells of origin, and express luminal cytokeratin 8/18 [45]. Luminal A subtype is either ER positive or progesterone receptor (PR) positive but is negative for HER2. Luminal B subtype can be classified as ER positive or PR positive and is positive for HER2 [46]. The prognosis for luminal A is better than luminal B and typically responds more effectively to selective estrogen receptor modulators, such as tamoxifen [45].

Estrogens have been implicated in breast cancer; however, the mechanism of action still remains unclear. One theory suggests that the mechanism is dependent on the activation of the ER. Estrogen induces breast cancer through stimulation of cellular proliferation, resulting in more opportunities for accumulation of genetic damages leading to carcinogenesis [47]. Another possible mechanism of action may be through the metabolism of estrogen, which may induce oxidative stress and play a key role in mammary cancer development [48,49]. 17β-Estradiol and estrone are continuously interconverted by 17β-estradiol hydroxysteroid dehydrogenase (or 17β-oxidoreductase) and are the two major endogenous estrogens. The carbon position of the estrogen molecules that are hydroxylated differs among various tissues in the body and each reaction is probably catalyzed by various CYP enzymes. For example, estrogen may be metabolized by CYP 1A1 to form 2-hydroxyestradiol (2-OHE2) or by CYP 1B1 to form 4-hydroxyestradiol (4-OHE2). These catechols may be methylated by a phase II enzyme, catechol o-methyltransferase (COMT), and excreted out of the body [50]. The 2-OHE2 metabolite is rapidly methylated by COMT, while the 4-OHE2 metabolite is methylated more slowly and thus highly genotoxic [49]. If catechol estrogens are not conjugated (mostly 4-OHE2), it may lead to the formation of semiquinones and subsequently quinones, both of which are electrophiles capable of covalently binding to nucleophilic groups on DNA which may form DNA-adducts [47]. The protective phase II enzyme, NAD(P)H dehydrogenase, quinone 1 (NQO1), catalyzes the reduction of quinones back to catechol estrogens [51]. 

Under normal conditions, reactive oxygen species (ROS) or RNS are neutralized by detoxifying and antioxidant enzymes [43]. Oxidative stress and/or electrophilic stress during redox cycling of catechol estrogens could contribute to nuclear factor (erythroid-derived 2)-like 2 (Nrf2) activation. The estrogen metabolites, 4-OHE1, 4-OHE2, and 2-OHE2 were capable of activating Nrf2, while estradiol did not [52]. This suggests that a catechol structure is required for activation of Nrf2. Estrogen metabolites may exert DNA mutations from ROS or DNA mutations which may lead to the accumulation of genomic alterations essential for mammary tumorigenesis [47]. In one study, 49 women without breast cancer were observed with larger amounts of 2-OHE2 than 4-OHE2 [53]. The 28 women with breast carcinoma expressed 4-OHE2 levels that were 3.5 times more abundant than 2-OHE2 [53]. This supports the finding that estrogen and its metabolites, mainly 4-OHE2, may be carcinogenic agents in breast epithelial cells [53]. 

### 2.2. Human Epidermal Growth Factor Receptor 2 (HER2)

HER2 amplification and overexpression has been reported in 18-25% of human breast cancers [54]. HER2 positive breast cancer can be characterized as HER2 positive, negative for ER, and poor differentiation [55]. The prognosis for HER2 positive is worse than luminal breast cancer. HER2 positive breast cancer may be treated with monoclonal antibodies such as trastuzumab (binds to domain IV on the HER2 receptor); however, there are HER2 positive tumors that are resistant to trastuzumab treatment [55]. Other treatments include monoclonal antibody pertuzumab (binds to domain II of the HER2 receptor) [56], trastuzumab antibody conjugated with mertansine (DM1), which is internalized and exerts its cytotoxic effects inside the cell [57], tyrosine kinase inhibitors [58], and HSP90 inhibition which leads to proteasomal degradation [59]. 

HER2 is a member of the epidermal growth factor (ErbB) family of transmembrane receptors which are potent mediators of normal cell growth and development [60]. The ErbB family is classified as a tyrosine kinase receptor and consists of EGFR (HER1), ERBB2 (HER2), ERBB3 (HER3), and ERBB4 (HER4). The structure consists of an extracellular domain at which the ligand binding occurs, the α-helical transmembrane segment, and the intracellular protein tyrosine kinase domain [61]. ErbB receptors normally exist as inactive monomers until a ligand initiates a conformational change to induce dimerization with another receptor. HER2 is unique in the fact that it already possesses an active tyrosine kinase domain and has no direct ligand while HER3 lacks an intrinsic tyrosine kinase activity and cannot form homodimers with itself [62]. The HER2-HER3 heterodimer is considered the most potent and active ErbB dimer [63,64,65]. HER2 signaling leads to oncogenic cell survival and proliferation through the MAPK pathway [66]. HER3 can directly bind to the p85 subunit of PI3K to stimulate the PI3K-Akt pathway while EGFR and HER2 have additional activation steps by binding to the adaptor proteins GRB2 (growth factor receptor bound 2) and GAB1 (GRB2-associated binding protein 1) [67]. Thus, the HER2-HER3 dimer leads to the MAPK pathway to stimulate angiogenesis, proliferation, and PI3K-Akt pathway to promote cell survival, suppression of apoptosis, and cell cycle control [66]. 

### 2.3. Basal-Like

Basal-like subtype is characterized by ER and HER2 negativity, high expression of basal stratified epithelial cytokeratins 5, 6, and 17, and expression of proliferation-related genes [45,68]. The prognosis of basal-like tumors is poor, with frequent mutations in *TP53* [69]. BRCA1 mutations are also generally basal-like breast tumors [68,69]. The incidence of basal-like breast cancer may be increased by both race and age, where premenopausal African American women developed basal-like tumors (39%) compared to postmenopausal African American women (14%) and non-African American women (16%) [70]. In addition, microarray analysis revealed that younger patients of any ethnicity tend to form basal-like tumors over other types [69,70]. 

## 3. Cellular Events and Molecular Targets in Breast Cancer

Each subtype of breast cancer responds differently to current treatments and therapy. To date, there is limited *in vitro*, *in vivo*, and human data which connect individual tocopherols for prevention or treatment for each subtype of breast cancer. Chemoprevention is an approach to prevent cancer before a series of genetic and epigenetic events establish which otherwise could lead to malignancies. Thus, prevention of breast cancer is essential, and the success of prevention strategies depends on understanding the molecular mechanism of breast cancer initiation and progression. The mechanisms of anti-cancer activity of tocopherols have been investigated for many years [39,71,72] and can be summarized as follows: (a) inhibition of ER (b) increasing peroxisome proliferator activated receptor γ (PPARγ) expression and activity, (c) induction of Nrf2, (d) antioxidative and anti-inflammatory activities, and (e) induction of apoptosis [28,39,43,72,73]. 

### 3.1. Estrogen Receptor (ER)

ER is a nuclear receptor that stimulates cell growth and proliferation [48]. The ER is a ligand-activated transcription factor that, when bound to estrogen, induces a conformational change that allows dimerization and binding to estrogen response element sequences. There are two known ER receptors: ERα and ERβ. The DNA binding domain of the two different receptors is highly homologous while the ligand binding domain is 60% homologous [74]. ERα and ERβ are both present in breast tissue, but the ratio of ERα to ERβ is increased in breast tumors [74]. The role of ERβ in breast tumorigenesis is not well understood. Some studies have shown that activation of ERβ in breast cancer cell lines inhibits cell growth, and the dimerization of ERβ with ERα silences the growth-promoting effects of ERα [74,75]. 

Vitamin E has been shown to inhibit ER-positive cell proliferation and work as antagonists of estrogen signaling in MCF-7 and T47D breast cancer cells [73]. MCF-7 cells were treated with γ-TmT, and the expression of ERα was down-regulated [7]. In mammary tumors, ERα mRNA and protein levels were down-regulated by the treatment of γ-TmT [7]. Administration of γ-TmT reduced ERα mRNA and protein levels in hyperplastic mammary tissues in estrogen-treated ACI rats, while mRNA levels of ERβ were increased [76]. Furthermore, dietary γ-TmT decreased circulating levels of E_2_ in the serum, suggesting that γ-TmT may modify the response to estrogen [76]. 

### 3.2. Peroxisome Proliferator Activated Receptor γ (PPARγ)

Belonging to the nuclear hormone receptor superfamily, PPAR comprises of 3 subtypes (α, γ, and δ) which are ligand-regulated transcription factors [77]. PPARγ is known to be involved in fatty acid uptake and transport and acts to control inflammation by inducing apoptosis and inhibiting cell proliferation cell survival [78,79]. PPARγ signaling is connected to the inhibition of inflammatory markers (COX-2, cytokines, and inducible nitric oxide synthase), PI3K/Akt pathway, and angiogenesis while inducing CDK inhibitors, differentiation and apoptosis markers in cancers [79]. Particularly in breast cancer, stimulation of PPARγ increases the degradation of cell cycle genes (Cyclin D1), interferes with estrogen receptor signaling, and NF-κB signaling cascades [80,81]. 

When ligand-activated, PPARγ forms a heterodimer with the retinoid X receptor [78]. A known PPARγ ligand is troglitazone [27], and the chromanol ring of tocopherol is structurally similar. Recently it was thought that tocopherols might function as a PPARγ ligand because of this structural resemblance, but it was shown that γ-tocopherol does not directly bind to PPARγ [81]. Instead, γ-tocopherol induces the formation of 15-*S*-hydroxyeicosatetraenoic acid, an endogenous PPARγ ligand [81]. 

Tocopherols, with γ-tocopherol displaying the strongest activity, increased mRNA and protein levels of PPARγ in colon cancer cells [27] and transcriptional activity in keratinocytes cell line [82] (Table 1). In MCF-7 and T47D breast cancer cells, γ-TmT, γ-tocopherol, and more strongly δ-tocopherol enhance the transactivation of PPARγ [7]. Comparable to the finding in the *N*-methyl-*N*-nitrosourea (NMU)-induced breast cancer model in Sprague-Dawley rats [7], PPARγ was increased at both the protein and mRNA level in the mammary gland of ACI rats when treated with γ-TmT while ERα expression was decreased [76]. Since PPARγ transactivation can be suppressed by ERα binding to the PPAR response element [83], the inhibition of ERα expression by tocopherols may result in the activation of PPARγ. Thus, tocopherols may indirectly activate PPARγ, and possibly through this pathway may interfere with ERα expression, inhibit cell cycle progression and induce apoptosis to prevent breast cancer. 

**Table 1:** Tocopherols induce PPARγ levels.

Tocopherol	Cell type/Cancer model	Result	References
γ-Tocopherol	Colon cancer cells (SW 480)	↑ PPARγ mRNA and protein level	[27]
γ-Tocopherol	Keratinocytes cells (NCTC 2544)	↑ PPARγ mRNA levels	[82]
γ-TmT, γ-Tocopherol, δ-Tocopherol	Breast cancer cells (MCF-7 and T47D)	↑ PPARγ transactivation	[7]
γ-TmT	NMU-induced mammary tumors in female Sprague-Dawley rats	↑ PPARγ mRNA and protein level	[7]
γ-TmT	Estrogen-induced mammary hyperplasia in female ACI rats	↑ PPARγ mRNA and protein level	[76]

### 3.3. Nuclear Factor (Erythroid-Derived 2)-Like 2 (Nrf2)

Nrf2 is a transcription factor that is a key regulator of cellular antioxidant and detoxification enzymes [84]. Initially, Nrf2 activity is inhibited when bound to kelch-like-ECH-associated protein 1 (KEAP1) in the cytoplasm and is marked for degradation through the proteasomal pathway [84]. Under oxidative stress or chemopreventive agents, KEAP1 undergoes covalent modification which allows the release and the consequential activation of Nrf2 [84,85,86]. As a result, Nrf2 translocates into the nucleus, dimerizes with small Maf proteins, and binds to the antioxidant-responsive element (ARE) to stimulate gene expression of antioxidant enzymes (thioredoxin, superoxide dismutase[SOD], catalase, glutathione peroxidase, and heme oxygenase-1[HO-1]), and phase II detoxification enzymes (glutathione s-transferases[GST], UDP-glucuronosyltransferases, sulfotransferases, and NQO1) [43,84,85,86,87]. As a result, these detoxifying and antioxidant enzymes protect cells from neoplastic transformation by maintaining oxidative stress homeostasis [43,88]. A loss of Nrf2 may lead to a decrease in cellular defense against oxidative stress which may result in tumorigenesis [89]. 

In human retinal pigment epithelial cells, pretreatment with α-tocopherol inhibited ROS generation, increased Nrf2 expression, and, induced phase II enzymes (glutamate cysteine ligase, NQO1, HO-1, GST, and SOD) [90] (Table 2). The expression of Nrf2 was suppressed in prostate tumors [91], and treatment with γ-TmT upregulated the expression of Nrf2 and detoxifying enzymes, and inhibited tumor development in TRAMP mice [43,91]. We recently demonstrated that when estrogen-treated ACI rats were administered γ-TmT diet, the protein expression level of Nrf2 was increased in the mammary gland and liver, and phase II enzymes were increased in the liver [92]. The mRNA expressions of phase II detoxifying enzymes were induced in the mammary gland and liver by γ-TmT treatment. This may indicate that γ-TmT induces the transcription of Nrf2-ARE-target genes and exhibits protective defense against estrogen induced oxidative stress. 

**Table 2:** Tocopherols induce Nrf2 and related antioxidant enzymes.

Tocopherol	Cell type/Cancer model	Result	References
α-Tocopherol	Human retinal pigment epithelial cells (ARPE-19)	↑ Nrf2 protein levels, ↑ glutamate cysteine ligase, NQO1, HO-1, GST, SOD	[90]
γ-TmT	Prostate carcinogenesis in TRAMP male mice	↑ Nrf2 protein levels, ↑ GSTm1, UGT1A1, HO-1, catalase, SOD, glutathione peroxidase,	[43]
γ-TmT	Estrogen-induced mammary hyperplasia in female ACI rats	↑ Nrf2 protein levels	[92]

### 3.4. Cell Proliferation and Apoptosis

Apoptosis is defined as programmed cell death with distinct morphological and biochemical changes [93,94]. During the earlier stages, the apoptotic cell shrinks in volume and the nuclear DNA condenses, while the cellular membrane remains intact [94,95]. Apoptotic bodies are formed and the tightly packed organelles leave the cell through “budding” [96]. There are two distinct apoptotic pathways: extrinsic and intrinsic [97]. Caspases have proteolytic activity and are able to cleave proteins. There are ten major caspases with three main sub groups: initiators (−2, −8, −9, and −10), effectors (−3, −6, and −7), and inflammatory (−1, −4, and −5) [98,99]. 

In breast, colon, lung, and prostate cancer cell lines, γ-tocopherol was shown to be more effective at inhibiting cell growth than α-tocopherol [7,25,26,100]. Our *in vitro* data showed that treatment with γ-TmT, γ-, and δ-tocopherol inhibited cell proliferation in MCF-7 breast cancer cells in a dose-dependent manner, while α-tocopherol did not [7]. In addition, a colony growth inhibition assay utilizing MDA-MB-435 breast cancer cells showed that γ- and δ-tocopherol showed potential to inhibit colony formation, whereas α‑tocopherol was not active [39]. 

γ-Tocopherol has been shown to induce apoptosis in breast, colon, and prostate cancer cells [26,100,101,102,103] (Table 3). Yu *et al.* showed that apoptosis was induced by δ-tocopherol in MCF-7 and MDA-MB-435 breast cancer cells [102]. Furthermore, γ-tocopherol, but not α-tocopherol, induced cleaved-caspase 8 and 9 in MDA-MB-435 human breast cancer cells [103]. In one xenograft model, when treated with γ-tocopherol, tumor growth was inhibited, and TUNEL assay determined that there was an increase in apoptotic cells [22]. γ-Tocopherol and to a greater extent, δ-tocopherol, were shown to inhibit tumor growth more strongly than α-tocopherol in a lung xenograft model, while α-tocopherol did not [42].

**Table 3:** Tocopherols inhibit cell proliferation and induce apoptosis.

Tocopherol	Cell type/Cancer model	Result	References
γ-Tocopherol	Prostate cancer cells (LNCaP and PC-3) and lung cancer cells (A549)	↓ Proliferation	[101]
γ-Tocopherol and combination of γ-Tocopherol and δ-Tocopherol	Prostate cancer cells (LNCaP)	↑ Apoptosis	[101]
γ-Tocopherol	Colon cancer cells (SW480, HCT-15, HCT-116, HT-29)	↓ Proliferation, ↑ Apoptosis	[100]
γ-Tocopherol	Prostate cancer cells (LNCaP)	↓ Proliferation, ↑ Apoptosis	[101]
δ-Tocopherol	Breast cancer cells (MCF-7 and MDA-MB-435)	↑ Apoptosis	[102]
γ-Tocopherol	Breast cancer cells (MCF-7 and MDA-MB-435) and murine 66cl-4	↓ Proliferation, ↑ Apoptosis	[103]
γ-Tocopherol	Bresat cancer MDA-MB-231 xenograft in nu/nu mice	↑ Apoptosis	[22]
γ-Tocopherol, δ-Tocopherol	Lung cancer H1299 xenograft in nu/nu mice	↑ Apoptosis	[42]
γ-TmT	NMU-induced mammary tumors in female Sprague-Dawley rats	↓ Proliferation	[104]
γ-TmT	NMU-induced mammary tumors in female Sprague-Dawley rats	↑ Apoptosis	[7]
γ-Tocopherol, δ-Tocopherol, γ-TmT	NMU-induced mammary tumors in female Sprague-Dawley rats	↑ Apoptosis	[105]
γ-TmT	Estrogen-induced mammary hyperplasia in female ACI rats	↓ Proliferation, ↑ Apoptosis	[76]

*In* *vivo* models showed that mammary tumor growth and burden was decreased by γ-TmT diet [7,104]. Proliferating cell nuclear antigen (PCNA) was decreased in mammary hyperplasia [76] and in mammary tumors when administered γ-TmT [104]. Administration of γ-TmT increased the levels of cleaved-caspase 3 increased in mammary hyperplasia [76] and in mammary tumors [7]. Furthermore, γ-TmT and individual tocopherols were administered to Sprague-Dawley rats which were induced with NMU carcinogen; treatment with γ-TmT, γ-, and δ-tocopherol decreased PCNA levels while increased the levels of cleaved-caspase 3 in mammary tumors, whereas α-tocopherol was not active [105]. At high doses, tocopherols may induce DNA damage leading to apoptosis. There is the possibility of tocopherols, especially α-tocopherol, to act as a pro-oxidant to create ROS or RNS.

### 3.5. Cyclooxygenase-2 (COX-2) and Anti-Inflammatory Activities

COX-2 is an inducible prostaglandin synthase which is upregulated by growth factors, tumor promoters, and cytokines [106], and responsive to several oncogenes, such as HER2 [107,108]. In inflamed and neoplastic tissues, an increase in prostaglandin synthesis is detected [107]. Around 40% of aggressive human breast cancers are associated with high levels of COX-2 which correlates with large tumor sizes, high proliferation rates, and metastases [108]. Celecoxib, a COX-2 inhibitor, was fed to HER2/neu transgenic mice and found that there was a 50% reduction in mammary prostaglandin E_2_ (PGE_2_) levels and delayed tumor onset [109]. 

Tocopherols are known antioxidants and anti-inflammatory agents, and γ-tocopherol is more effective in inhibiting the activity of COX-2 and trapping reactive nitrogen species than α-tocopherol (Table 4) [23,28,29,30,31,110]. In addition, γ-tocopherol was shown to reduce PGE_2_ synthesis in macrophages and human epithelial cells [28], and the inhibitory effect was due to the decrease of COX-2 activity [28,111]. In our study, serum levels of PGE_2_ and 8-isoprostane, a marker of oxidative stress, were reduced when estrogen-induced ACI rats were treated with γ-TmT, and COX-2 levels decreased in the mammary gland when treated with dietary γ-TmT [76]. γ-TmT treatment may reduce inflammation in an estrogen-induced model of mammary hyperplasia and tumorigenesis. 

**Table 4:** Tocopherols decrease COX-2 and RNS.

Tocopherol	Cell type/Cancer model	Result	References
γ-Tocopherol	Carrageenan-induced inflammation in Wistar male rats	↓ RNS, ↓ PGE_2_, ↓ LTB_4_, ↓ TNF-α	[29]
γ-Tocopherol	Macrophages (RAW264.7) and human epithelial cells (A549)	↓ COX-2, ↓ PGE_2_	[28]
γ-Tocopherol	Zymosan-induced acute peritonitis in male Fischer 344 rats	↓ RNS	[30]
γ-Tocopherol	Human plasma	↓ RNS, ↓ peroxynitrite	[31]
γ-Tocopherol, δ-Tocopherol	Human epithelial cells (A549)	↓ COX-2	[111]
γ-TmT	Estrogen-induced mammary hyperplasia in female ACI rats	↓ COX-2, ↓ PGE_2_, ↓ 8-isoprostane	[76]

## 4. Studies on Tocopherols and Human Cancers

### 4.1. Case-Control and Cohort Studies

There are several case-control, cohort, and intervention studies on vitamin E and human cancers, but our main focus will address breast cancer. Numerous case-control studies utilized vitamin E and 11 studies found a risk reduction [112,113,114,115,116,117,118,119,120,121,122], however, 13 studies did not find an association with breast cancer incidence (Table 5) [123,124,125,126,127,128,129,130,131,132,133,134,135]. In the Shanghai Breast Cancer Study, they suggest that vitamin E supplement may reduce the risk of breast cancer among women who have low dietary intake [122]. To date, 12 cohort studies did not find any relation between vitamin E and prevention of breast cancer risk (Table 6) [136,137,138,139,140,141,142,143,144,145,146,147]. In one cohort study, the European Prospective Investigation into Cancer and Nutrition (EPIC) trial observed that vitamin E did not reduce breast cancer risk, but there was a weak risk reduction in post-menopausal women [145]. While investigating vitamin supplement during breast cancer treatment and survival, Nechuta *et al.* determined that vitamin E supplementation in the first 6 months after diagnosis may reduce risk of mortality and recurrence [148]. 

**Table 5:** Case-control studies of vitamin E and breast cancer risk.

Study	Population	Year	Case/Control ^a^	Intake or blood levels	Relative risk (95% CI) for highest *vs.* lowest level	Conclusion
[124]	Canada	1989-1993	223/85	Serum or adipose tissue levels of α-T: levels were not specified	Serum α-T: 0.85 (0.45-1.59)	No association
					Adipose tissue α-T:1.34 (0.73-2.47)	
[125]	US	1976-1998	969/969	Serum α-T or γ-T: levels were not specified	Serum α-T: 0.79 (0.57-1.08)	No association
					Serum γ-T: 0.96 (0.71-1.30)	
[126]	US	1975-1994	244/244 (1974 Study)	Serum α-T: 0.91-1.40 mg/dL; 0.99-1.65 mg/dL	Serum α-T: 0.94 (0.52-1.73); 0.67 (0.28-1.62)	No association
			115/115 (1989 Study)	Serum γ-T: 0.15-0.32 mg/dL; 0.13-0.34 mg/dL	Serum γ-T: 0.70 (0.40-1.23); 0.80 (0.33-1.93)	
[127]	US	1975-1993	64/64	Serum α-T: 1.31 mg/dL	α-T: 0.46 (0.23-0.64)	No association
				Serum γ-T: 0.25 mg/dL	γ-T: 0.53 (0.32-0.69)	
[113]	India		Pre-M: 28/23	Serum α-T: 38 *vs.* 25 μmol/L	Serum α-T: *P* < 0.05	Risk reduction
			Post-M: 29/19	Serum γ-T: 30 *vs.* 25 μmol/L	Serum γ-T: *p* < 0.02	
[129]	US		27/28	Serum α-T: ≤20.5 ~ ≥35 μmol/L	Serum α-T: 0.76 (0.10-5.75)	No association
				Serum γ-T: ≤2.12~ ≥7.573 μmol/L	Serum γ-T:0.31 (0.04-1.93)	
[130]	Greek		Pre-M: 270/505	Vit E: <5.2 ~ ≥8.6 IU/day	Pre-M: 0.50 (0.25-1.02)	No association
			Post-M: 550/1041		Post-M: 0.85 (0.53-1.36)	
[114]	Finish		Pre-M: 119/324	Vit E: ≤7 ~ >13 mg/day	0.5 (0.2-1.0)	Risk reduction
[115]	Uruguay		400/405	Vit E: 4.7 ~ 9.7 mg/day	0.4 (0.26-0.62)	Risk reduction
[131]	Italian		Pre-M: 989/841	Vit E: <8.5 ~ 11.7 mg/day	Pre-M: 1.27 (0.9-1.78)	No association
			Post-M: 1577/1745		Post-M: 1.16 (0.92-1.46)	
[132]	US	1977-1989	105/203	Serum α-T: ≤21.6 ~ ≥31.3 μmol/L	1.2 (0.5-2.8)	No association
[116]	Italy		Pre-M: 988/843	Vit E: levels were not specified	Pre-M: 0.8 (0.7-1.0)	Risk reduction
			Post-M: 1572/1742		Post-M: 0.75 (0.6-0.9)	
[117]	US		297/311	α-T: <6 ~ ≥11 mg/day	0.55 (0.34-0.88)	Risk reduction
[112]	US		Pre-M without family history: 224/251	α-T: ≤6.3 ~ >10.4 IU/day	0.5 (0.2-1.0)	Risk reduction
[118]	US		Post-M: 313/349	Vit E: 11 *vs.* 5.4 mg/day (median)	0.4 (0.2-0.9)	Risk reduction
[119]	Malaysia		57/139	Vit E: 6.1 *vs.* 6.9 mg/day (mean)	2.12 (1.00-4.21)	Risk reduction
[128]	South Korea	2004-2006	362/362	Vit E: 10.6 *vs.* 11.2 mg/day	0.66 (0.41-1.08)	No association
[120]	Switzerland	1993-1999	289/442	Vit E: 9.4-18.1 mg/day	0.49 (0.35-0.71)	Risk reduction
[121]	Italy	1991-1994	2569/2588	Vit E: 7.21-13.43 mg/day	0.75 (0.6-0.9)	Risk reduction
[123]	Germany	1998-1999	310/353	Vit E: 7.1-12.7 mg/day	1.08 (0.58-2.03)	No association
[122]	China	1996-1998 and 2002-2004	3454/3474	Vit E: levels not specified	Low supplemental Vit E: 0.7 (0.5-1.0)	Risk reduction
					High supplemental Vit E: 1.2 (0.9-1.6)	
[133]	US	1999-2004	1498/1559 (Non-Hispanic white) 763/877 (Hispanic)	α-T: 108-224 mg/day	α-T: 0.87 (0.73-1.03)	No association
				β-T: 0.3-0.4 mg/day	β-T: 1.10 (0.89-1.36)	
				γ-T: 15.9-19.4 mg/day	γ-T: 1.13 (0.89-1.44)	
				δ-T: 2.94-3.59 mg/day	δ-T: 1.10 (0.89-1.35)	
[134]	Denmark	1993-1997	418/394	Dietary Vit E:	Dietary Vit E: 1.13 (0.61-2.10)	No association
				4.30-14.8 mg/day	Supplemental Vit E: 1.00 (0.96-1.03)	
				Supplemental Vit E: 0.94-78.23 mg/day		
[135]	South Korea	1999-2000	224/250	Dietary Vit E: 6.26-12.71 mg/day	Dietary Vit E: 0.71 (0.39-1.27)	No association

^a^ Pre-menopausal (Pre-M) or postmenopausal (Post-M) women.

Previously, detailed assessments revealed that vitamin E (α-tocopherol) supplements did not protect against breast cancer [149,150]. Recently, Fulan *et al.* performed a meta-analysis on 38 studies between vitamin E and breast cancer [151]. For case-control studies, dietary vitamin E and total vitamin E reduced breast cancer risk by 18% and 11%, respectively [151]. When the cohort studies were pooled with the case-control studies, dietary vitamin E and total vitamin E both became nonsignificant [151]. Thus, a conclusion remains elusive between breast cancer and vitamin E. The term “vitamin E” is used loosely, and a distinction in these case-control and cohort studies need to clarify which variant of vitamin E is utilized. 

**Table 6:** Cohort studies of vitamin E and breast cancer risk.

Study	Population	Year	Case/Control ^a^	Intake or blood levels	Relative risk (95% CI) for highest *vs.* lowest level	Conclusion
[136]	Canada	1982-1987	519/1182	α-T: <3 *vs.* >7 mg/day	α-T: 1.05 (0.65-1.70)	No association
[137]	Sweden	1987-1990	1271/59036	Vit E: 9.3 *vs.* 3.8 mg/day (median)	0.83 (0.6-1.14)	No association
[138]			Pre-M: 784/53938	Vit E: 10 *vs.* 5 IU/day (median)	0.81 (0.64-1.02)	No association
[139]	Netherlands		Post-M: 650/62573	Vit E: 19.8 *vs.* 6.9 mg/day (median)	1.25 (0.85-1.85)	No association
[140]	Finland		88/4697	Vit E: levels were not specified	1.08	No association
[141]	US	1986	570/21782	Vit E: 10 *vs.* 5 mg/day	0.81 (0.64-1.02)	No association
[142]	US	1976-1982	1439/89494	Dietary Vit E: <3.9 ~ ≥24.1 IU/day	Dietary Vit E: 0.90 (0.77-1.06)	No association
				Supplemental Vit E: 600 *vs.* 0 IU/day	Supplemental Vit E: 1.01 (0.69-0.49)	
[143]	Canada		325/628	Vit E: ~18 IU/day (median)	1.32 (0.85-2.05)	No association
[144]	US	1980-1987	Post-M: 344/18586	Vit E: <4.3 ~ ≥9.3 mg/day	0.86 (0.61-1.21)	No association
[145]	Europe	1992-2000	7502/334493	Vit E: 5.4-19.5 mg/day	0.92 (0.77-1.11)	No association
[146]	US	1993-1998	2879/81926	Dietary Vit E: 6.2-9.4 mg/day	Dietary Vit E: 1.03 (0.91-1.17)	No association
				Supplemental Vit E: 0-424 mg/day	Supplemental Vit E: 1.01 (0.90-1.14)	
[147]	US	1991-1999	Pre-M: 714/90655	Vit E: 7-59 mg/day	Vit E: 1.13 (0.89-1.43)	No association
				Dietary Vit E: 6-10 mg/day	Dietary Vit E: 1.17 (0.92-1.50)	

^a^ Pre-menopausal (Pre-M) or postmenopausal (Post-M) women.

### 4.2. Intervention Studies

The Alpha-Tocopherol, Beta-Carotene (ATBC) Cancer Prevention Study examined the prevention of lung and other cancers with supplementation of all-racemic-α-tocopherol acetate (50 mg/day) and β-carotene (20 mg/day) daily, which did not have an effect on lung or colorectal cancer [152,153]. However, the ATBC study found that males supplemented with α-tocopherol acetate (50 mg daily) had 32% lower prostate cancer incidence and 41% reduction in prostate cancer deaths [154]. The Physicians’ Health Study II gave supplements of 400 IU of α-tocopherol every other day or 500 mg of vitamin C daily and concluded that neither vitamin E nor C reduced the risk of prostate cancer [155]. The Selenium and Vitamin E Cancer Prevention Trial (SELECT) administered selenium (200 μg/day) and *all rac*-α-tocopheryl acetate (400 IU/day) and revealed that selenium or vitamin E, alone or in combination, did not prevent prostate cancer [156]. These previous clinical and epidemiological studies have been primarilyutilized α-tocopherol, and not a mixture of tocopherols or other variants of tocopherols for chemoprevention [37,38,39,40]. 

There have been 3 breast cancer randomized controlled trials (RCT), which administered supplemental natural-source vitamin E (either 400 IU or 600 IU), and concluded that there was no overall benefit of vitamin E supplementation [37,157,158]. Only one RCT specified using the variant α-tocopherol [158], but in most cases, the studies do not identify which variant of vitamin E was utilized. Thus, epidemiological evidence between vitamin E and breast cancer is limited and inconsistent [41]. There are four tocopherols and four tocotrienols that comprise vitamin E, each which differ in chemical structure, bioavailability, and activity. Results will remain inconclusive unless the specific variant is identified for each study. 

## 5. Conclusion

α-Tocopherol has been investigated over many years, while data are lacking for γ- and δ-tocopherols. A γ-enriched mixture of tocopherol is commonly found as a by-product of corn oil and should also be explored. The status of tocopherol as a chemopreventive agent remains unclear due to inconsistent results. In previous case-control and cohort studies, the term vitamin E may be vague, with few studies specifying which variant is utilized. A distinction needs to be addressed to determine the efficacy of each tocopherol variant and its chemopreventive activity. It has been suggested that γ-TmT, γ-tocopherol, and more recently δ-tocopherol may contribute to inhibiting tumor formation. Possible mechanism of actions in inhibiting breast cancer could be: inducing PPARγ expression and as a result reducing the expression of ERα, inducing Nrf2 which consequently reduces inflammation and oxidative stress, and inhibiting cell proliferation while inducing apoptosis. Further investigation is warranted with γ-TmT, γ- and δ-tocopherol in human prevention trials. 
